# Relationship Between "Geosminophilia" (Liking the Smell of Earth) and Pica in Patients With Iron Deficiency

**DOI:** 10.7759/cureus.50460

**Published:** 2023-12-13

**Authors:** Muhammet Özbilen

**Affiliations:** 1 Internal Medicine, Ordu University Faculty of Medicine, Ordu, TUR

**Keywords:** geosminophilia, olfactory signs, geosmin, symptom, smell of earth, pica, iron deficiency

## Abstract

Background: Iron deficiency is a critical global health issue. One of the intriguing manifestations linked to iron deficiency is the presence of pica. The physiological relationship between the senses of smell and taste suggests that pica may be accompanied by an inclination towards the smell of earth (geosmin). The smell of geosmin is distinct and generally recognized as a pleasing scent. The objective of this study is to explore this potential association. The sign of liking the smell of earth will now be referred to as "geosminophilia", for the first time, in this study.

Methods: Patients aged 18 years and above who visited the Iron Deficiency Outpatient Clinic between September 2021 and August 2022 were chosen for this cross-sectional study. The dataset excluded those with ferritin concentrations over 100 mcg/L and transferrin saturation over 45%. Demographics, presence of pica, history of geosminophilia, and laboratory findings were documented in physical and digital patient data. The descriptive statistics, chi-square test, and independent group comparison tests were performed.

Results: With a mean age of 38.7±11.7 years, the study sample comprised 280 patients, of which 263 were female and 17 were male. Ninety-six patients (34.3%) had geosminophilia, while 29 patients (10.4%) had pica. The only factors that significantly distinguished the groups based on the presence of geosminophilia were gender (p=0.04) and age (p=0.02). Using the chi-square test to compare the presence of pica and geosminophilia, a statistically significant relationship was identified (p<0.001). Additionally, 72.4% of patients who presented with pica had geosminophilia. Pica was absent in 96% of patients who did not have geosminophilia. A total of 93.8% (15/16) of pica cases with geophagia subtype exhibited the presence of geosminophilia. A significant correlation (r=0.27, p<0.001) was also observed between geosminophilia and pica.

Conclusion: In iron deficiency patients, geosminophilia and the presence of pica may accompany each other. Therefore, geosminophilia has the potential to be a new symptom of iron deficiency.

## Introduction

Iron, which is widely recognized as a fundamental component in the origin of life [1], is the most prevalent element in the earth's mass [2]. Deficiency of this element is a common condition that impacts a significant portion of the world's populations, contributing to the global burden of disease worldwide [3]. Iron deficiency is linked to various medical, mental, and organ-related complications [4,5].

Certain patients with iron deficiency exhibit a strange inclination to instinctively seek out soil, which serves as an inorganic reservoir of iron in living organisms. This phenomenon is commonly referred to as geophagia, which is a very common form of pica, or the ingestion of non-food substances [6]. There is a prevailing view that the inorganic nutrient requirements, particularly iron, associated with pica are predominantly fulfilled by soil [7].

On the other hand, it is recognized that certain essential neuronal enzymes implicated in the sense of smell contain inorganic iron, which is necessary for these enzymes to function [8]. Therefore an analogous compulsion to consume inorganic substances can likewise be exhibited through an overwhelming inclination for olfactory stimulation [9].

Regardless of the illness, certain individuals possess a profound liking for a particular odor: the smell of earth or geosmin [10]. This olfactory phenomenon, also known as petrichor, which refers to the characteristic smell of dry soil following rainfall, can be attributed, at least in part, to the release of a chemical odorant called geosmin by soil-dwelling bacteria [11]. While the enjoyment of the smell of earth elicits a pleasurable and pleasant experience, there exists a lack of research exploring its potential medicinal implications.

The importance of scent with regard to a substance or food that is to be consumed is beyond dispute. Hence, the extent to which patients experiencing pica due to iron deficiency developed an affinity for the smell of earth was a subject of investigation in this research.

In the context of this study, the term "geosminophilia" will be used as a new word to refer to the tendency to like the smell of earth.

## Materials and methods

Study design and patient selection

This was a cross-sectional, retrospective study. Patients who were admitted to the Iron Deficiency Outpatient Clinic at Ordu University Training and Research Hospital, Türkiye, during the period from September 2021 to August 2022 were included in the study. The study was approved by the Ethics Committee of Ordu University (approval number: 2023/291) and it was conducted in accordance with the principles outlined in the Declaration of Helsinki.

The Iron Deficiency Outpatient Clinic at our hospital exclusively caters to those who have received a medical diagnosis of iron deficiency, as indicated by its name. Hence, it can be inferred that each patient who was admitted to the outpatient clinic either had an ongoing iron deficiency condition or had recently undergone iron replacement therapy.

The major exclusion criteria were: patients below the age of 18 and patients whose hospital records contained significant data gaps. Additionally, patients were excluded from the dataset if their serum ferritin concentration surpassed 100 mcg/L or if their transferrin saturation level exceeded 45%. The main reason for applying this criterion was to specifically eliminate acute infectious or inflammatory conditions, as well as disorders associated with iron overload rather than iron deficiency.

Data type

The patient data included in the sample were obtained from both physical and digital patient files, and the dataset was produced using Excel (Microsoft Corporation, Redmond, Washington, United States). Initially, demographic data types (age and gender) were documented. As part of the regular routine of the Iron Deficiency Clinic, each incoming patient is interviewed on a comprehensive range of symptoms. Patients are queried regarding the presence of the pertinent complaint and/or symptom. The responses obtained are classified as either "present" or "absent." In the same manner, responses to the presence of pica and liking for the smell of earth are also collected. The binary responses (absent/present) in the dataset precisely represented the observations regarding geosminophilia and the presence of pica, which served to elucidate the investigating hypothesis. Finally, the main biochemical biomarkers, including serum ferritin, transferrin saturation (TSAT), hemoglobin (HGB), and mean corpuscular volume (MCV), were extracted from the medical records of the patients. In addition, a categorical variable was added to the dataset to denote the existence of anemia. To accomplish this, the HGB cut-off values recommended by the World Health Organization (WHO) of 12 g/dL for women and 13 g/dL for males were selected [12].

Statistical analyses

The data were analyzed using version 2.4.8 of the Jamovi program [13]. During the initial phase, the sample's mean age and gender distribution were computed. A p-value less than 0.05 was considered statistically significant. Following this, the rates and quantities of individuals who had geosminophilia and pica were computed. Following the completion of these descriptive statistics, a t-test for independent groups was conducted on continuous data categorized by the presence of geosminophilia. Then, using the chi-square test, the relationship between the presence of geosminophilia and three other categorical variables, namely gender, anemia, and pica, was investigated. 

New medical term generation

The term geosminophilia has been coined for the first time in this paper. The synthesis of this word was as follows: The two distinct Greek roots "ge" (earth) and "osme" (smell) are the basis of the word geosmin. The suffix "-philia" signifies love or tendency for a particular subject. Thus, the term "geosminophilia" was generated by combining geosmin and -philia with o used as a combining vowel [14].

## Results

A sample of 280 patients who met the inclusion criteria for the study were identified. The sample consisted of 263 females, accounting for 93.9% of the total, and 17 males, representing 6.1% of the total. The mean age of the entire sample was 38.7±11.7 years. The mean age of female patients was 37.6±10.7 years, whereas the mean age of male patients was 55.1±14.7 years. A summary of the results obtained from the biochemical analysis is presented in Table 1. Upon examination of the mean values of these biochemical data, it becomes evident that the sample exhibits the presence of iron deficiency anemia. However, the biochemical results did not exhibit a statistically significant disparity based on gender (p>0.05).

**Table 1:** Laboratory results of the study sample.

Variables	n	Mean ±SD	Reference values
Hemoglobin	280	10.7 ±2.05	11.9-14.6 g/dL
Mean corpuscular volume	280	77.5 ±10.33	82.9-98.0 fL
Transferrin saturation	266	10.7 ±8.46	20-45%
Ferritin	270	12.0 ±12.90	13-150 mcg/L

Table 2 displays the frequencies and percentages pertaining to the presence of geosminophilia and pica. Based on the findings, it was revealed that geosminophilia was present in approximately one-third of the patients (34.3%). On the other hand, pica was identified in 10.4% of the patients. 

**Table 2:** Results for the presence of pica and geosminophilia among all patients.

Signs	+/-	Frequency	Percentage of Total
Pica	+	29	10.4%
	-	251	89.6%
Geosminophilia	+	96	34.3%
	-	184	65.7%

A comparison was conducted between independent groups that were generated based on the existence of geosminophilia. The groups were compared according to age, HGB, MCV, TSAT, and ferritin. The results indicated a significant difference between the groups only in terms of age (t (278)=2.33, p=0.02). The mean age of patients with geosminophilia (36.42±10.12) was significantly lower than those without this finding (39.84±12.35).

The categorical variable representing the existence of anemia consisted of 197 patients (70.4%) who were classified as anemic, while 83 patients (29.6%) were classified as non-anemic. The chi-square test did not reveal any statistically significant difference between the presence of geosminophilia and the presence of anemia (X2(1, N=280)=0.022, p=0.881). The rate of anemia in individuals with geosminophilia (69.8%, n=67) was similar to the rate of anemia in patients without geosminophilia (70.7%, n=130).

A significant difference was found when examining the association between geosminophilia and gender, as indicated by the results of a chi-square test (X2(1, N=280)=4.07, p=0.044). It was shown that only 11.8% (n=2) of males exhibited geosminophilia, compared to 35.7% (n=94) of women.

The presence of geosminophilia and pica was compared with the chi-square test (Table 3) and a high level of significance was found (X2(1, N=280)=20.9, p<0.001). It was noteworthy that 72.4% of the patients who presented with pica also expressed a liking for the smell of earth. Approximately 96% of individuals who did not express a liking for the earthy smell did not have pica, which is another remarkable statistic.

**Table 3:** Results of the comparison of pica and geosminophilia by chi-square test.

Geosminophilia	Pica		Total
	Absent	Present	
Absent	176	8	184
% within row	95.7%	4.3%	100.0%
% within column	70.1%	27.6%	65.7%
Present	75	21	96
% within row	78.1%	21.9%	100.0%
% within column	29.9%	72.4%	34.3%
Total	251	29	280
% within row	89.6%	10.4%	100.0%
% within column	100.0%	100.0%	100.0%

A classification of pica categories revealed that among the patients analyzed, 16 (55.2%) had geophagia, five (17.2%) ate coffee or coffee grounds, and four (13.8%) had pagophagia. Fifteen of the 16 patients (93.8%) who presented with geophagia also exhibited geosminophilia.

An additional result was obtained regarding the relationship between geosminophilia and pica using the Spearman correlation test and a correlation of moderate significance was observed between the two signs (r(278)=0.273, p<0.001).

## Discussion

The present study marks the first mention of "geosminophilia" as a disease finding in the medical literature. Again, the current research has made a significant contribution to the field of olfactory preferences, specifically concerning geosminophilia, which is the liking for the smell of earth. The recognition of geosminophilia as a unique phenomenon highlights the importance of investigating atypical facets of human behavior and sensory perception.

The observation that 34.3% (n=96) of the patients in the study liked the smell of earth and that 72.4% of those with pica also had geosminophilia appears to be a remarkable finding. Geosminophilia suggests that the senses of smell and taste may interact [15], as evidenced by the fact that all but one (93.8%) of the patients with geophagia also had geosminophilia. The absence of pica in around 96% of patients without geosminophilia provides further support for the notion that the odor-eating relationship may hold true under pathological conditions [16], as demonstrated by this result.

An additional prominent result was that the prevalence of geosminophilia was considerably higher among females. This may be ascribed to the superiority of women over males in sensory abilities including identification, memory, odor perception, and discrimination [17]. Similarly, the observation that the liking for the smell of earth was notably greater in the younger group can be ascribed to the decrease in olfactory sensitivity that occurs with advancing age [18].

The study analyses indicate a statistically significant link and correlation between geosminophilia and pica, suggesting a potential physiological and/or pathological relationship between these two phenomena. This observation suggests the potential existence of a shared fundamental element, specifically a causal relationship associated with iron deficiency.

Several studies have discussed this eating and olfactory psychomotor behavior. In a study conducted among a cohort of pregnant women, it was shown that 53.6% of the participants reported smell as the precipitating factor for engaging in pica behavior [19]. Again, three cases reported in 2019 indicate an association between iron deficiency anemia and increased smell sensitivity [20]. Additionally, the presence of an elevated sense of smell in response to specific inorganic substances led to the diagnosis of iron deficiency anemia in a patient, as documented in a case report from 2021 [21]. Another study used rats to experimentally demonstrate how olfactory behavior is affected by iron deficiency. Rats who had an iron deficiency exhibited a considerably extended duration of sniffing in comparison to the control group [22].

Limitations

The nature of the earthy smell does not have a generally agreed-upon characteristic; rather, individual preferences and perceptions may vary, in addition to cultural factors that could potentially influence this odor's reception. The study's data regarding the presence of geosminophilia were presented in a binary "yes/no" format. Results that are more meaningful and/or interpretable could be obtained using a rating scale, such as a five-point Likert scale. Finally, the participants included in this retrospective study were either preexisting iron deficiency patients or patients who had recently had treatment for iron deficiency. Thus, this study was unable to conduct a statistical comparison regarding the prevalence of geosminophilia between patients with iron deficiency and those who were healthy.

## Conclusions

Geosminophilia, similar to pica observed in patients who have iron deficiency, has a notable connection with soil, which serves as a primary source of the iron element. In both terminology and clinical relevance, this is the first contribution to the literature on the connection of liking the smell of earth with pica. Geosminophilia is observed in nearly all patients with geophagia, and the variation in these findings according to age and gender provides additional evidence for the interaction between smell and taste in iron deficiency. This suggests that liking the smell of earth may be a novel symptom of iron deficiency. It is necessary to conduct comparisons with healthy groups in order to validate the pathological implications of this finding.
